# A Flexible Multifunctional Sensor Based on an AgNW@ZnONR Composite Material

**DOI:** 10.3390/ma17194788

**Published:** 2024-09-29

**Authors:** Hao Lv, Xue Qi, Yuxin Wang, Yang Ye, Peike Wang, Ao Yin, Jingjing Luo, Zhongqi Ren, Haipeng Liu, Suzhu Yu, Jun Wei

**Affiliations:** 1 Shenzhen Key Laboratory of Flexible Printed Electronics Technology, Harbin Institute of Technology (Shenzhen), Shenzhen 518055, China; 20s055025@stu.hit.edu.cn (H.L.); qixue@hit.edu.cn (X.Q.);; 2 School of Materials Science and Engineering, Harbin Institute of Technology (Shenzhen), Shenzhen 518055, China; 3 State Key Laboratory of Advanced Welding and Joining, Harbin Institute of Technology (Shenzhen), Shenzhen 518055, China

**Keywords:** multifunctional sensor, AgNW@ZnONRs, transparent ultraviolet photodetectors, flexible, strain gauges

## Abstract

A multifunctional sensor comprising flexible and transparent ultraviolet (UV) photodetectors (PDs) with strain gauges based on Ag nanowire (AgNW)@ZnO nanorods (ZnONRs) was fabricated using a cost-effective, simple, and efficient method. High-aspect ratio silver nanowires were synthesized using the polyol method. An AgNW@ZnONR composite was formed via the hydrothermal method to ensure the multifunctional capability of the flexible sensors. After refining the process parameters, the size of the ZnO nanorods was decreased to fabricate pliable multifunctional sensors using AgNW@ZnONRs. At a deposition of 0.207 g of AgNW@ZnONRs, the sensor achieves its maximum switching ratio and fastest response time under conditions of 2000 μW/cm^2^ UV optical power density. With a t_on_ (rise time) of 2.7 s and a t_off_ (fall time) of 2.3 s, the ratio of I_on_ to I_off_ current is 1151. Additionally, the sensor’s maximum optical current value correlates linearly with UV light’s power density. The maximum response current increased from 222.5 pA to 588.1 pA, an increase of 164.3%, when the bending angle was increased from 15° to 90° for the sensor with a deposition of 0.276 g of AgNW@ZnONRs. There was no degradation in the response of the sensors after 10,000 bending cycles, as they have excellent stability and repeatability, which means they can meet the requirements of wearable sensor applications. Therefore, there is great potential for the practical application of multifunctional AgNW@ZnONRs in flexible sensors.

## 1. Introduction

With the rapid advancement of technology and specific practical needs, the drive toward sensor miniaturization, integration, or multifunctionality on a single sensor platform has intensified. The burgeoning field of flexible multifunctional sensors, capable of sensing diverse signals, is increasingly being recognized as a pivotal research area [1,2,3].

Zinc oxide (ZnO) stands out as a biocompatible material with significant promise for ultraviolet (UV) sensing applications due to its wide bandgap of 3.37 eV at room temperature and high exciton binding energy of 60 meV [4,5]. A critical factor in developing UV sensors based on ZnO is the realization of low electron density and high electron mobility before UV exposure. The correlation between lower electron densities and reduced dark currents is well established, significantly enhancing sensor sensitivity and accuracy [6,7,8]. Electron depletion in ZnO can be effectively facilitated by surface-adsorbed oxygen or by decoration with metal nanoparticles possessing high work functions, particularly in nanostructures with a large specific surface area [9,10]. This makes ZnO nanowires, nanorods, or nanoparticles ideal for fabricating flexible sensors [5,11,12].

Furthermore, the integration of ZnO nanostructures with other nanomaterials has been established as a strategy that can not only help enhance the sensing capabilities of sensors but also help to achieve multifunctionality. However, previous studies often involved mere mixing of ZnO nanostructures with other materials, which resulted in poor material interfacing and suboptimal electrical signal transmission, adversely affecting sensor performance [13,14,15].

To address these challenges, our study utilized a two-step hydrothermal method to directly grow ZnO nanorods on silver nanowires, creating AgNW@ZnONR composite materials [15,16]. This composite serves as the functional layer, with polydimethylsiloxane (PDMS) employed as a flexible substrate. The resultant flexible multifunctional sensor developed in this study is capable of detecting multiple signals, such as those derived from ultraviolet light and bending, showcasing its applicability in a wide range of scenarios. This innovative approach not only enhances the electrical connectivity between the composite materials but also significantly boosts the sensor’s overall performance and reliability.

This research contributes to the field by highlighting the efficacy of using advanced composite materials in sensor development and reporting the successful application of these materials in real-world scenarios, thereby pushing the boundaries of sensor technology.

## 2. Experimental Section

This work utilized the polyol method to synthesize silver nanowires, adjusting variables such as the types and concentrations of ionic additives, the amount of reducer, and reaction durations to produce high-purity silver nanowires of varied sizes. Further, AgNW@ZnONR composite materials were prepared using a hydrothermal method, and a flexible multifunctional sensor was fabricated on a PDMS substrate.

### 2.1. Experimental Materials

The experiment employed several high-purity reagents sourced from reputable manufacturers. Polyvinylpyrrolidone (PVP), silver nitrate (AgNO_3_), ethylene glycol, sodium chloride, and sodium bromide, all analytical grade, were provided by Aladdin Reagents (Shanghai, China) Co., Ltd. Copper chloride and sodium hydroxide of similar purity were sourced from the same company. Benzoic acid and acetone, essential for the process, were obtained from Sinopharm Chemical Reagent Co., Ltd. (Shanghai, China), while XiLong Scientific supplied anhydrous ethanol and isopropanol (Shanghai, China). Deionized water, crucial for various dilution and cleaning steps, was of the ultrapure variety and obtained from Watsons (Shanghai, China). Essential compounds for the synthesis of zinc oxide nanostructures, such as zinc acetate dihydrate and zinc nitrate hexahydrate, were obtained from Aladdin, as was hexamethylenetetramine. The sensor’s flexible substrate comprises DC184 optical adhesive-grade PDMS and its curing agent, both procured from Dow Corning (Midland, MI, USA).

### 2.2. Experimental Equipment

We utilized a number of precision instruments to ensure accurate experimental results. For mixing and stirring, a multi-point magnetic stirrer (model HL6A) from Jiangsu Kexi Instrument Co., Ltd. (Jiangsu, China) and a constant-temperature heating magnetic stirrer (model DF-101S) from Shanghai Lichen Bangxi Instrument Technology Co., Ltd. (Shanghai, China) were used. Weight measurements were taken using an electronic analytical balance (model FA1204B) from Hengji Instruments Co., Ltd. (Jiangsu, China). The synthesis processes required controlled centrifugation, provided by a benchtop high-speed centrifuge (model TDL-5-A) from Shanghai Anting Scientific Instrument Factory (Shanghai, China), and temperature regulation was achieved with an electric constant-temperature convection oven (model BJ-1400X) from Hefei Kejing Material Co., Ltd. (Hefei, China). The cleaning of materials was facilitated by the use of an ultrasonic cleaner (model JP-040) from Shanghai Sheyan Technology Co., Ltd. (Shanghai, China). Precise volumes of liquids were handled using an adjustable single-channel pipette (20–200 μL) from Dalong Instruments (Beijing, China). Hydrothermal synthesis reactions were carried out in a 100 mL reactor from Xi’an Changyi Instruments Co., Ltd. (Xi’an, China). The spin coating process for film deposition involved the use of a spin coater (model VTC-50A) from MTI Corporation (Richmond, CA, USA). The electrical properties of the nanowires were assessed using a four-point probe conductivity meter (model ROOKO FT-347) from Rooko Instruments Co., Ltd. (Jiangsu, China), as well as an electrochemical workstation (model CHI760D) from Shanghai Chenhua Instruments Co., Ltd. (Shanghai, China). Current measurements were conducted with an ammeter (model KEITHLEY-6221) from Keithley Instruments Inc. (Cleveland, OH, USA), and UV response tests were performed using a UV LED point light source (270 nm/365 nm) from Shenzhen Heshengbang Technology Co., Ltd. (Shenzhen, China). Material characterization was performed using a field-emission scanning electron microscope (Crossbeam350) from Zeiss (Oberkochen, Germany).

## 3. Fabrication of AgNW@ZnONR-Based Flexible Multifunctional Sensors

### 3.1. Surface Morphology of AgNW@ZnONRs

The synthesis of AgNW@ZnONR composite materials was conducted using a two-step hydrothermal method [17]. This process enabled the cultivation of densely packed zinc oxide nanorods on surfaces of high-aspect ratio silver nanowires, designed to maximize surface interactions between the components [18,19]. Silver nanowires (AgNWs) were chosen for their unique one-dimensional structure, which provides a high surface area, excellent electrical conductivity, and tunable optical properties. These characteristics are attributed to the polyol reduction method used in their synthesis, which favors anisotropic growth, resulting in nanowires rather than other particle shapes.

**Figure 1:**
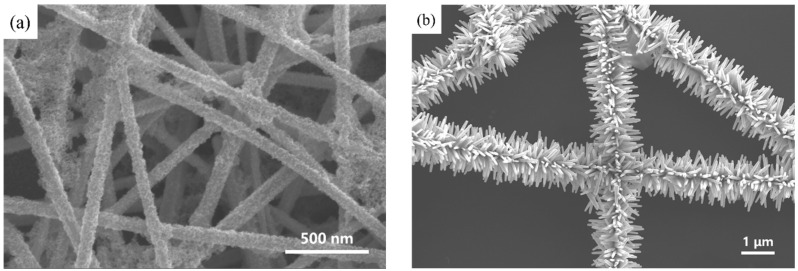
SEM micrographs of the synthesized composite materials: (**a**) AgNW@ZnONPs; (**b**) AgNW@ZnONRs.

Initially, the silver nanowire solution was combined with a ZnO seed solution under hydrothermal conditions. Given the similarity in lattice spacing between ZnO and Ag, ZnO nanoparticles started to adsorb onto the surface of the silver nanowires [20,21]. This adsorption was facilitated by high-temperature agitation, leading to the formation of tens of nanometer-sized ZnO particles. These particles aggregated to create a uniform nanoparticle coating layer on the silver nanowires, forming the AgNW@ZnONP composite material (Figure 1a). The purity of the AgNWs plays a crucial role in achieving this uniform coating and is supported by several factors, including the synthesis method, the morphological uniformity of the nanowires (as observed in Figure 1a), and the absence of visible contaminants. The smooth, defect-free structure of the AgNWs further underscores their high purity and suitability as a template for ZnO growth.

**Figure 2:**
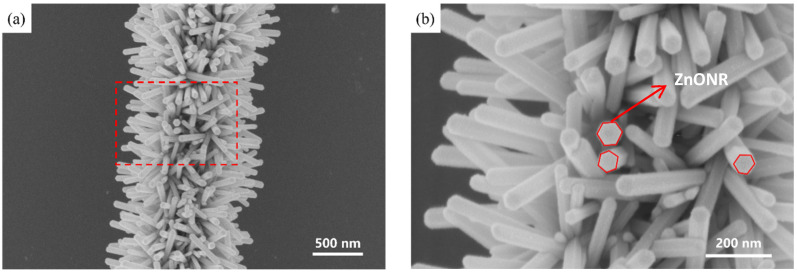
SEM plot of AgNW@ZnONRs under the initial parameters: (**a**) morphology representation of AgNW@ZnONRs; (**b**) local enlarged view of AgNW@ZnONRs.

Following the initial reaction, the AgNW@ZnONP solution was introduced into a secondary ZnO growth solution. The continued deposition of ZnO at high temperatures allowed for the radial growth of one-dimensional nanorods from the surfaces of the silver nanowires, culminating in the AgNW@ZnONR composite material. This material exhibited a distinctive “test tube brush” morphology, where ZnO nanorods uniformly enveloped the AgNW surfaces without any exposed silver nanowires, as illustrated in Figure 1b and further detailed in Figure 2a. Further analysis revealed that the ZnO nanorods were typically hexagonal prism-shaped, approximately 60 nm in diameter, and around 500 nm in length, having uniform dimensions (Figure 2b).

The growth of ZnO nanorods was influenced by multiple factors, including the concentration of the seed solution, the amount of AgNWs added, the concentration of the growth solution, the hydrothermal reaction temperature, and reaction time. These parameters significantly affected the size, morphology, and uniformity of the ZnO nanorods. Research on ZnO-based UV and gas sensors has shown that the specific surface area of ZnO critically affects the sensor’s sensitivity and responsiveness [22,23,24]. Increasing the specific surface area enhances the contact area between ZnO and oxygen, reducing the electron density in the absence of UV exposure, which is beneficial for achieving high sensitivity and responsiveness in ZnO sensors [25,26].

**Figure 3:**
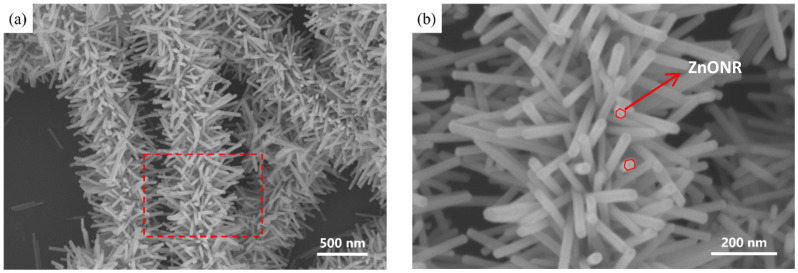
The SEM plot of AgNW@ZnONRs after optimizing the parameters: (**a**) morphology representation of AgNW@ZnONRs; (**b**) local enlarged view of AgNW@ZnONRs.

In this work, the process parameters were optimized by systematically adjusting the concentrations of the seed and growth solutions, as well as the reaction temperature. This optimization led to the synthesis of an AgNW@ZnONR composite material with an enhanced specific surface area, as illustrated in Figure 3. The resulting ZnO nanorods exhibited lengths ranging from 200 to 300 nm and diameters ranging from 20 to 30 nm. These parameters facilitated the fabrication of flexible multifunctional sensors.

### 3.2. Construction of Flexible Multifunctional Sensors

AgNW@ZnONRs were dispersed in isopropanol (IPA) to create solutions at concentrations of 0.069 g/mL, 0.138 g/mL, 0.207 g/mL, and 0.276 g/mL. Each solution was prepared to ensure homogeneous particle distribution, which is critical for achieving uniform sensor performance. A 0.5 mL volume of polydimethylsiloxane (PDMS) was then drop-cast onto a clean glass substrate measuring 50 mm × 20 mm × 1 mm. The PDMS was spread evenly and heated on a hotplate at 80 °C for 5 min to reach a semi-cured state. This intermediate curing stage was crucial for maintaining the right viscosity for subsequent material deposition without fully setting the polymer.

Following the semi-curing process, 1 mL of each AgNW@ZnONR/IPA solution was evenly applied to the surface of the PDMS. The quantity of the solution used varied with the concentration, resulting in different amounts of AgNW@ZnONR composite material being deposited—specifically 0.069 g, 0.138 g, 0.207 g, and 0.276 g. This careful control of the deposition process is essential for tuning the functional properties of the sensors, such as their thickness and conductivity.

**Figure 4:**
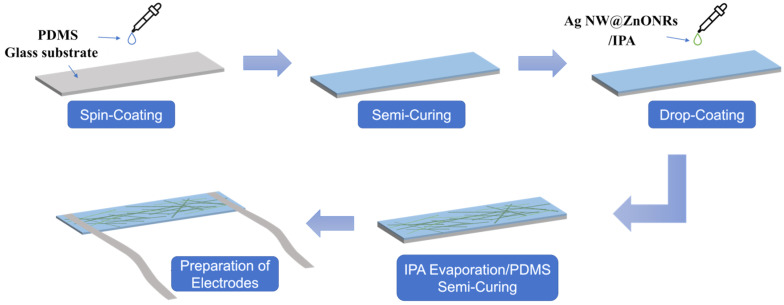
Preparation flow diagram of AgNW@ZnONR-based flexible multifunctional sensor.

After allowing the IPA to fully evaporate, the glass substrate was placed back on the hotplate and heated at 80 °C for an additional 20 min to completely cure the PDMS, thus forming a robust flexible film. The film was subsequently detached and cut into segments of 50 mm × 10 mm. Conductive silver tape was attached to both ends of these segments to serve as electrodes. To ensure robust electrical connectivity, a liquid conductive silver paste was applied between the electrodes and the AgNW@ZnONRs. The assembly was then heated to 100 °C for 10 min to cure the paste, solidifying the electrical connections and enhancing the overall sensor structure, as depicted in Figure 4.

The structural propensity of AgNW@ZnONRs to tangle and clump, coupled with their inherent hydrophobicity, poses challenges for deposition techniques such as spin coating. These characteristics can lead to delamination during practical application, potentially resulting in sensor failure. The use of semi-cured PDMS allowed the composite materials to be securely embedded within the PDMS surface layer, significantly enhancing the durability and reusability of the conductive layer during repeated usage and making it highly suitable for wearable flexible sensors.

**Figure 5:**
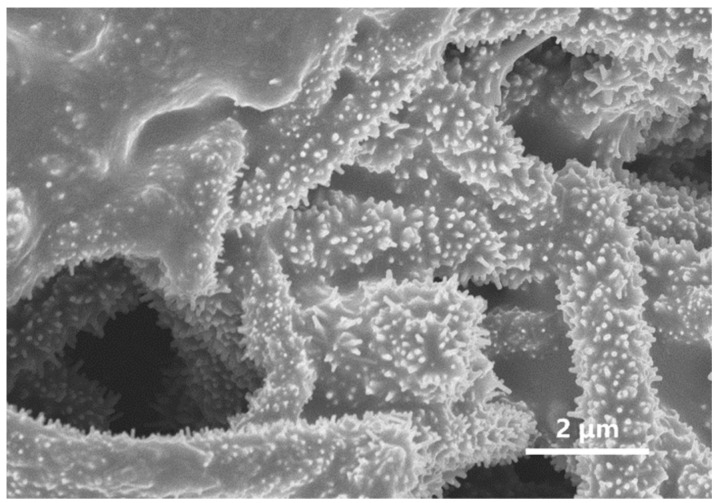
The SEM diagram of the AgNW@ZnONR conductive layer on the film surface.

The surface morphology of the AgNW@ZnONRs conductive layer on the film was further examined using scanning electron microscopy (SEM), which confirmed the uniformity and integrity of the sensor structure, as shown in Figure 5.

## 4. Results and Discussions

### 4.1. UV Response Testing

**Figure 6:**
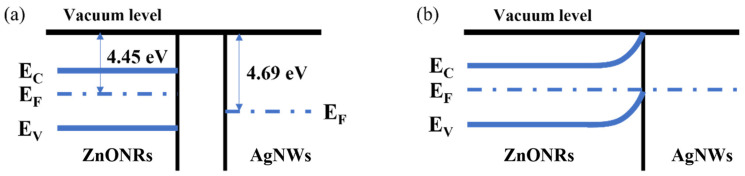
AgNW and ZnONR contact energy band. (**a**) Electron flow induces charge separation. (**b**) Formation of a space charge region and band bending.

**Figure 7:**
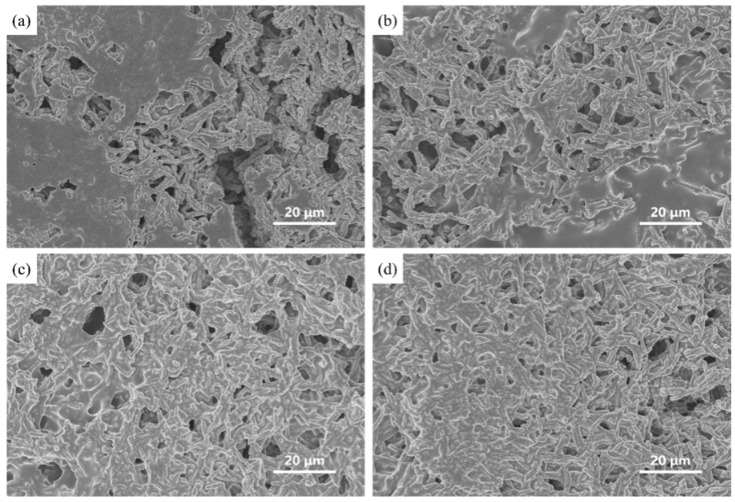
SEM diagram of different deposits of PDMS surface composites: (**a**) 0.069 g; (**b**) 0.138 g; (**c**) 0.207 g; and (**d**) 0.276 g.

Energy level diagrams illustrating the interaction between AgNWs and ZnONRs are presented in Figure 6. AgNWs have a work function of 4.69 eV, while ZnONRs exhibit a lower work function of 4.45 eV. Consequently, the Fermi level of ZnO (EFs) is higher than that of AgNWs (EFm), as shown in Figure 7a. Upon contact between AgNWs and ZnONRs, electrons flow from ZnONRs to AgNWs, resulting in a negative charge accumulation on the AgNWs surface and a positive charge on the ZnONRs surface. This charge transfer enhances the semiconductor potential of ZnO and reduces the potential of AgNWs. As the contact progresses, the gap between the metal and semiconductor narrows, increasing the density of the negative charge on the AgNW surface and the positive charge on the ZnONR surface [25,27]. Eventually, this leads to the formation of a positive space charge region on the surface of ZnO, which generates an internal electric field, causing energy band bending until equilibrium is reached, as depicted in Figure 7b [28,29]. This band bending introduces a surface barrier and forms a Schottky contact, creating a depletion layer [30].

Upon exposure to ultraviolet light, electrons in ZnO’s valence band absorb photons and transition to the conduction band, generating electron–hole pairs [31]. These carriers diffuse and drift under an external electric field, forming a photocurrent. The presence of a depletion layer due to the space charge region allows electrons to rapidly move from AgNWs towards ZnONRs, while the holes react with oxygen ions to form molecular oxygen. As the electron density on the ZnONRs side gradually increases, the space charge region narrows [32,33]. The thickness of the depletion layer swiftly reduces, the band bending dissipates, and the Fermi level of ZnONRs remains higher than that of AgNWs, enhancing carrier mobility and thereby increasing the photocurrent [34]. Upon removal of UV light, the potential difference due to the Fermi level discrepancy causes electrons to swiftly move back towards the AgNWs side, restoring the bent energy levels and accelerating the photocurrent recovery, thus shortening the recovery time [27].

The constructed sensors were tested under a 365 nm UV lamp, with the UV lamp’s power and the distance to the sensor adjusted to control varying UV light intensities. The sensor’s response was measured using an electrochemical workstation before and after exposure to different UV light intensities. The deposition amounts of the AgNW@ZnONR composite material on the sensor surface were set at 0.069 g, 0.138 g, 0.207 g, and 0.276 g, respectively. It was observed that at lower AgNW@ZnONR deposition amounts, a significant amount of PDMS covered the surface of the AgNW@ZnONRs, preventing the ZnO nanorods from contacting air. As the content of AgNW@ZnONRs increased, the covering effect of PDMS diminished, and more ZnO nanorods were exposed to the air.

For UV light sensors, the dark current (I_off_), photocurrent (I_on_), rise time (t_on_), and fall time (t_off_) are crucial metrics for assessing sensor performance. The current on/off ratio (I_on_/I_off_) indicates the UV response sensitivity, while the rise (response) time (t_on_) and fall (recovery) time (t_off_) reflect the UV sensor’s response speed. Here, the rise time is defined as the duration it takes for the response current to increase from 10% to 90% of the maximum photocurrent when exposed to UV light; similarly, the fall time is defined as the time it takes for the current to decrease from 90% to 10% of the maximum photocurrent after the UV light is turned off.

**Figure 8:**
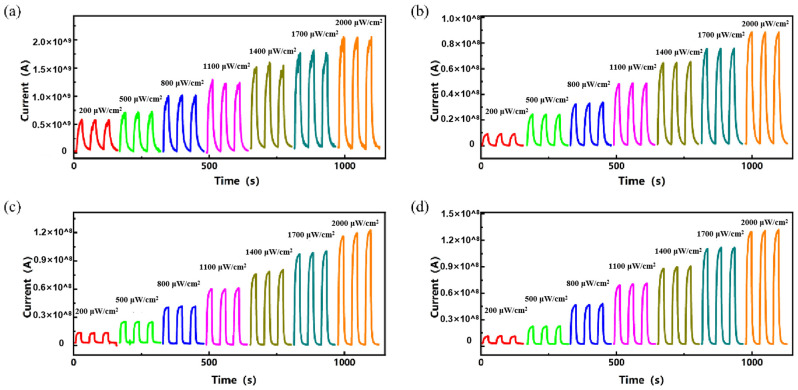
I-t curves of different deposition amounts in UV light response test: (**a**) 0.069 g; (**b**) 0.138 g; (**c**) 0.207 g; and (**d**) 0.276 g.

UV light sensors with AgNW@ZnONR composite material deposition amounts of 0.069 g, 0.138 g, 0.207 g, and 0.276 g were placed under the UV lamp and tested for their response performance to different UV light intensities at a bias of 1 V. The UV light power densities on the sensor surface were set at 200 μW/cm^2^, 500 μW/cm^2^, 800 μW/cm^2^, 1100 μW/cm^2^, 1400 μW/cm^2^, 1700 μW/cm^2^, and 2000 μW/cm^2^. Ultraviolet (UV) light irradiation was set for 20 s, followed by a shutdown of 30 s, resulting in a cycle duration of 50 s. The resulting current-time (I-t) curves are presented in Figure 8. As depicted in Figure 8a–d, the response current of the sensor increases with the rising power density of UV light. This enhancement is attributed to the excitation of more electrons from the valence band to the conduction band in ZnO under higher power densities, which increases the conductivity of zinc oxide and elevates the current. Additionally, under the same power densities, the response current slightly increases with the increased content of AgNW@ZnONRs, likely due to the increased conductive pathways provided by the greater amount of AgNW@ZnONRs, enhancing the overall conductivity of the AgNW@ZnONR conductive layer. As shown in Figure 8a, with an AgNW@ZnONR deposition of 0.069 g, the sensor’s response current primarily ranges between 10^−10^ A and 10^−9^ A. The highest on/off ratio and the fastest response times occur at a UV power density of 2000 μW/cm^2^, where the on/off ratio (I_on_/I_off_) is only 28.4, the rise time (t_on_) is 6.3 s, and the fall time (t_off_) is 7.4 s. It is also noted that the sensor’s dark current slightly increases with higher UV power densities. As illustrated in Figure 7a, this occurs because a substantial amount of ZnO nanorods, completely encapsulated by PDMS, retain a high electron concentration due to the inability of the electrons to combine with atmospheric oxygen. This results in a significant dark current when the sensor is not exposed to UV light, which, in turn, reduces the on/off ratio and extends the response time. In the case of a 0.138 g deposition of AgNW@ZnONRs, as shown in Figure 8b, the response current under UV irradiation was significantly enhanced compared to a deposition of 0.069 g. The dark current remains relatively stable. The highest on/off ratio and fastest response times also occur at a UV power density of 800 μW/cm^2^, reaching an Ion/Ioff ratio of 197.0, with a ton of 2 s and a toff of 4.2 s. Despite some AgNW@ZnONRs still being fully covered by PDMS, most outer layers of ZnO nanorods are exposed, leading to a notable increase in photocurrent and a reduction in dark current due to efficient recombination of surface-accumulated electrons with atmospheric oxygen, significantly enhancing the sensitivity and response time of the sensor. As depicted in Figure 8c, with an AgNW@ZnONR deposition of 0.207 g, the photocurrent further increases, ranging between 10^−9^ A and 10^−8^ A under UV irradiation. The dark current decreases to the 10^−12^ A range, which is advantageous for further enhancing the sensor’s sensitivity and response speed. The highest on/off ratio and fastest response times are observed at a UV power density of 2000 μW/cm^2^, where the I_on_/I_off_ ratio reaches 1151, the ton is 2.7 s, and the t_off_ is 2.3 s. At this stage, the outer layers of ZnO nanorods in AgNW@ZnONRs are almost entirely exposed to air. Given the greater electronegativity of oxygen compared to zinc oxide, the surface electrons of zinc oxide recombine with atmospheric oxygen, forming a depletion layer and promoting the diffusion and drift of photogenerated electrons towards the holes in zinc oxide, thereby significantly reducing the electron density in zinc oxide and subsequently lowering the dark current post-UV exposure. With an AgNW@ZnONR deposition of 0.276 g, as shown in Figure 8d, the photocurrent slightly increases under UV irradiation compared to a deposition of 0.207 g, but the increased deposition also leads to a higher dark current post-UV exposure, reaching the 10^−11^ A range. The highest on/off ratio and fastest response times are observed under a UV power density of 2000 μW/cm^2^, with an I_on_/I_off_ ratio of 589, a t_on_ of 3.2 s, and a t_off_ of 3.1 s. At this stage, even though the outer layers of AgNW@ZnONRs are well-exposed to air, the increased entanglement and accumulation of AgNW@ZnONRs following PDMS curing reduce the surface porosity of the sensor, limiting the interaction of the outermost AgNW@ZnONRs with atmospheric oxygen and hindering the reduction in dark current, thus leading to a decreased on/off ratio and slightly prolonged response time.

**Figure 9:**
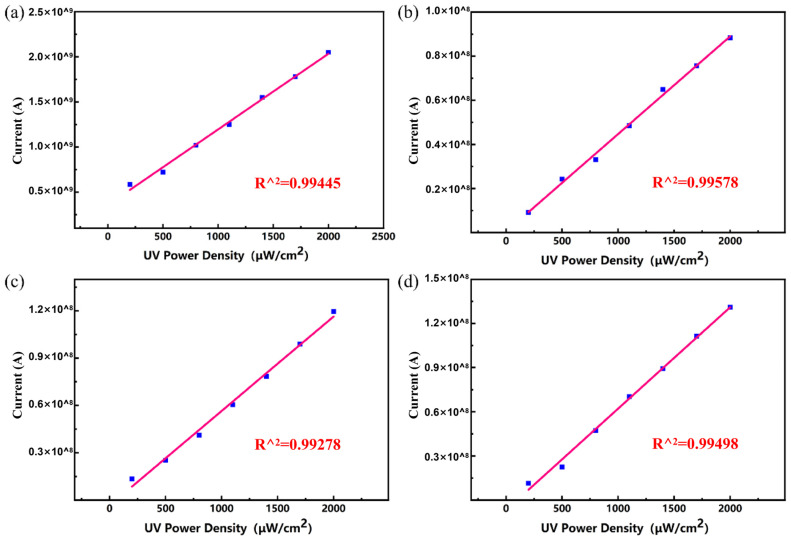
Linear fitting diagram of maximum photocurrent with different deposition amounts: (**a**) 0.069 g; (**b**) 0.138 g; (**c**) 0.207 g; and (**d**) 0.276 g.

Conversely, as shown in Figure 7a–c, the formation of a porous structure on the sensor’s surface post-PDMS curing allows the internal AgNW@ZnONRs and the surface ZnO nanorods to be fully exposed to air, ensuring substantial oxygen adsorption. This structure can significantly lower the sensor’s dark current, enhancing its UV detection sensitivity and response speed. It is known that the maximum photocurrent response increases with the rising UV power density across different AgNW@ZnONR depositions. A linear fitting of the maximum photocurrent for sensor depositions of 0.069 g, 0.138 g, 0.207 g, and 0.276 g under various UV power densities is illustrated in Figure 9. The results demonstrate a linear relationship between the maximum photocurrent and the UV power density, with excellent fitting outcomes indicated by R^2^ values above 0.99. This suggests that sensors based on AgNW@ZnONRs can be used to detect UV light, which means they can also sense and measure the power density of UV light, and that they could have promising applications in the development of wearable sensors.

### 4.2. Bending Response Testing

**Figure 10:**
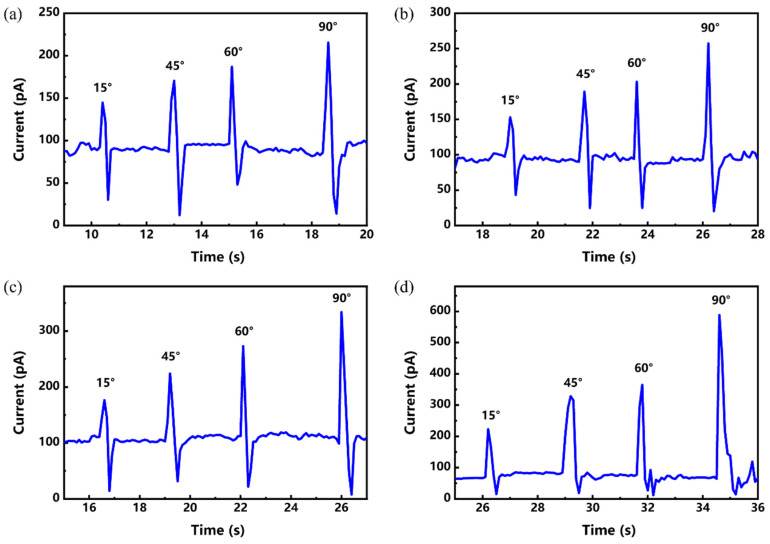
Bending response current of AgNW@ZnONR-based flexible sensors with different deposition amounts: (**a**) 0.069 g; (b) 0.138 g; (**c**) 0.207 g; and (**d**) 0.276 g.

**Figure 11:**
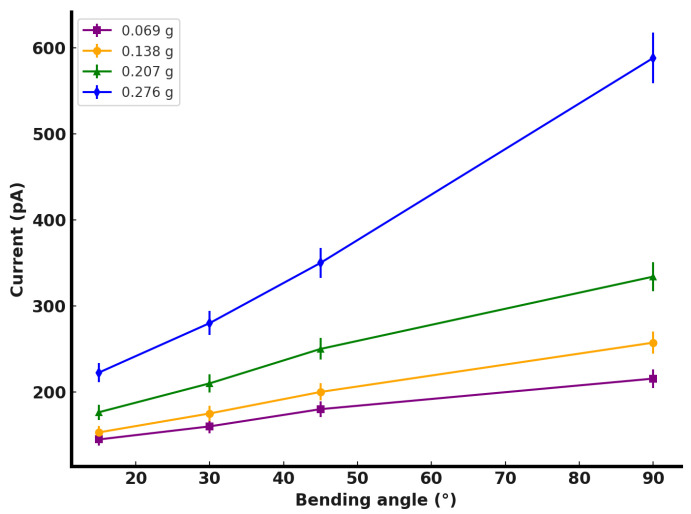
Relationship diagram of maximum response current–bending angle of sensors with different AgNW@ZnONR deposition amounts.

To investigate the mechanical signal detection capabilities of AgNW@ZnONR-based flexible multifunctional sensors and thereby demonstrate their versatility, dynamic bending response tests were conducted on four sensors with varying AgNW@ZnONR deposition amounts: 0.069 g, 0.138 g, 0.207 g, and 0.276 g. Each sensor was mounted on the fixtures of a tensile bending testing machine. A voltage of 5 V was applied, and the sensors were subjected to bending angles of 15°, 30°, 45°, and 90°. The resulting electrical currents generated during the bending process were recorded using an electrochemical workstation, and the results are shown in Figure 10. The relationship between the maximum response current and the bending angle is depicted in Figure 11.

As demonstrated in Figure 10, a sharp increase in current was observed at the moment of bending for sensors with different AgNW@ZnONR deposition amounts. Moreover, the maximum current value increased with the bending angle. At the same bending angle, the maximum response current increased with the quantity of AgNW@ZnONRs deposited. Importantly, for all four different deposition amounts of AgNW@ZnONR-based flexible sensors, the response time to bending deformation was exceptionally rapid, within 0.2 s, indicating a capability for a swift response to bending signals.

As illustrated in Figure 11, for sensors with AgNW@ZnONR deposition amounts of 0.069 g, 0.138 g, and 0.207 g, the increase in maximum response current from a bending angle of 15° to 90° was not significant. The current values rose from 144.8 pA, 153 pA, and 176.4 pA to 215.5 pA, 257.3 pA, and 334 pA, respectively, representing increases of 48.8%, 68.2%, and 89.3%. However, for the sensor with an AgNW@ZnONR deposition amount of 0.276 g, the maximum response current significantly increased from 222.5 pA at 15° to 588.1 pA at 90°, marking a 164.3% increase. This notable increase in electrical current upon bending is attributed to the piezoelectric effect of ZnO within the AgNW@ZnONR composite material. When the sensor undergoes bending deformation, the local regions subjected to tensile stress cause a separation of positive and negative charge centers in ZnO, generating an electric field that drives electron movement, thereby significantly increasing the sensor current. At lower deposition amounts, the piezoelectric effect generated during bending is less pronounced due to the lower ZnO content and the extensive covering by PDMS, which dissipates and absorbs most of the transmitted tensile stress, further reducing the stress experienced by ZnO and resulting in lower response currents. However, as the AgNW@ZnONR deposition amount increases, the quantity of ZnO significantly rises, enhancing the piezoelectric effect during bending. Despite being covered by PDMS, the reduction in PDMS between adjacent AgNW@ZnONRs allows more stress to be transmitted to ZnO under the same bending angles, thus generating significantly larger response currents during deformation.

### 4.3. Stability Testing

**Figure 12:**
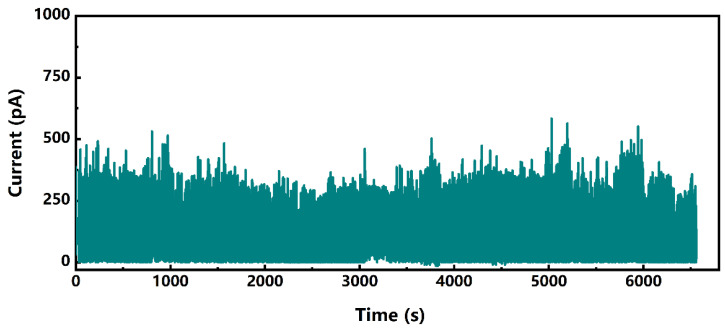
Results for a sensor that was bent at 90° for 10,000 cycle tests with a 0.276 g deposition amount.

To further assess the long-term stability and repeatability of the flexible multifunctional sensor’s performance, the sensor with the best performance from the bending response tests (AgNW@ZnONR deposition of 0.276 g) underwent a bending cycle test. The sensor was subjected to a bending angle of 90° for 10,000 cycles. The resultant current-time characteristic curve, as shown in Figure 12, indicates that the sensor exhibited good repeatability, with consistent response currents observed during each bending event. After enduring 10,000 bending cycles, the sensor’s bending response current still remained above 300 pA without significant degradation, demonstrating exceptional stability and repeatability.

Furthermore, after completing the 10,000 bending cycles, the sensor showed no signs of damage or deformation, indicating a prolonged lifespan suitable for wearable sensor applications. These results demonstrate the durability and consistent performance of AgNW@ZnONR-based flexible multifunctional sensors.

## 5. Conclusions

This work has successfully demonstrated the advanced capabilities of AgNW@ZnONR-based flexible multifunctional sensors, marking strides in the field of materials science and sensor technology. Our investigation reveals that these sensors not only exhibit robust mechanical flexibility but also superior sensitivity and rapid response to both UV and mechanical stimuli.

The incorporation of AgNW@ZnONR composite materials into the sensor architecture enabled distinct improvements in UV sensitivity and mechanical response behaviors. Specifically, the synthesis of these composites using a finely tuned hydrothermal process resulted in a sensor that combines the high electrical conductivity of AgNWs with the piezoelectric properties of ZnONRs. This hybrid material effectively translates mechanical deformations and UV light exposure into measurable electrical signals, offering a dual functionality that is seldom achieved with such efficiency in current sensor technologies. Moreover, the unique “test tube brush” structure of the ZnONRs increased the effective surface area for interaction with environmental stimuli, which also enhanced the electron mobility across the sensor interface. This structural innovation contributes significantly to the sensor’s quick electrical response and high sensitivity, and this is particularly noticeable under varying degrees of UV exposure and mechanical bending. The dynamic bending tests further delineated the sensors’ capacity to maintain structural integrity and performance after 10,000 bending cycles, underlining their durability and reliability for wearable applications. The ability of these sensors to withstand extensive mechanical flexing without degradation represents a breakthrough in the durability of wearable sensors. Additionally, the fast response times (within 0.2 s) to bending stress and the ability to precisely tune the sensor responses by adjusting the deposition thickness of AgNW@ZnONRs are notable. Such features highlight the sensors’ potential for real-time monitoring applications, where rapid and reliable feedback is crucial.

In conclusion, the AgNW@ZnONR-based sensors developed in this research embody a significant technological advancement due to their multifunctional capabilities, offering a promising avenue for future developments in smart wearable devices. Their innovative design, high sensitivity, rapid response, and proven durability position these sensors as a pivotal contribution to sensor technology, poised to help meet the rising demand for reliable, flexible, and multifunctional sensing platforms in various industrial, environmental, and health-related applications.

## Data Availability

All relevant data are within the paper.
